# High proportion of tuberculosis recent transmission in rural areas of Northeastern China: a 3-year prospective population-based genotypic and spatial analysis in Hinggan League, China

**DOI:** 10.1128/spectrum.00169-25

**Published:** 2025-07-11

**Authors:** Xichao Ou, Xiangchen Li, Shaojun Pei, Bing Zhao, Liping Feng, Chong Teng, Yewei Lu, Hanfang Zhu, Yang Zhou, Hui Xia, Zhengwei Liu, Xiaomeng Wang, Yanling Wang, Richard Anthony, Yanlin Zhao

**Affiliations:** 1 National Key Laboratory of Intelligent Tracking and Forecasting for Infectious Diseases, National Center for Tuberculosis Control and Prevention, Chinese Center for Disease Control and Prevention 12415, Beijing, China; 2 Key Laboratory of Precision Medicine in Diagnosis and Monitoring Research of Zhejiang Province, Hangzhou, Zhejiang, China; 3 School of Public Health, Peking University 12465, Beijing, China; 4 Department of Microbiology, Hinggan League Center for Disease Control and Prevention, Ulanhot, China; 5 Department of Tuberculosis Control and Prevention, Zhejiang Provincial Center for Disease Control and Prevention, Hangzhou, China; 6 Administration Office, Medical Insurance Service Center of Ulanhot City, Ulanhot, China; 7 Centre for Infectious Disease Control, National Institute for Public Health and the Environment, Bilthoven, the Netherlands; FIND, Geneva, Switzerland

**Keywords:** *Mycobacterium tuberculosis*, genotypic, spatial, whole-genome sequencing, transmission

## Abstract

Tuberculosis (TB) remains a significant public health challenge in China, particularly in rural areas like Hinggan League (HL), Inner Mongolia. Understanding the genetic diversity and transmission dynamics of *Mycobacterium tuberculosis* (MTB) strains is crucial for effective TB control. We conducted a prospective study from 2021 to 2023, sequencing 221 MTB isolates from HL. After quality control, 210 cases were analyzed. The genomic clustering rate was calculated to evaluate the level of recent transmission. Risk factors were identified by logistic regression analysis. Geospatial analysis was conducted with kernel density estimation. The majority of strains belonged to sub-lineage 2.2.1 in lineage 2 (L2), also known as the Beijing family (89.0%, 187/210), while the remainder belonged to lineage 4 (L4). L2 strains showed greater genetic similarity and shorter branch lengths compared with L4 strains. The overall drug resistance rate was 21.9%, with six multidrug-resistant TB (MDR-TB) and five pre-extensively drug resistant TB (pre-XDR-TB) cases identified. Almost half of the strains belonged to putative transmission clusters within 10 SNPs. Logistic regression analysis identified living in Jalaid Banner and being infected by L2 strains as significant risk factors for recent transmission. Spatial analysis identified spatial aggregation of TB cases in the eastern region of HL, with a hotspot for recent transmission in Jalaid Banner. The temporal distribution of TB cases in HL exhibited seasonal fluctuations, with diagnosis rates peaking in the first half of each year, and a notable increase in clustered cases in 2022. This study provides insights into the molecular epidemiology and transmission dynamics of TB in HL. Our results underscore the ongoing problem of TB transmission in rural settings, indicating the need for targeted interventions. These findings are vital for informing TB control strategies in HL and similar settings.

**IMPORTANCE:**

Tuberculosis (TB) remains a major public health problem in China. This study provides insights into the molecular epidemiology and transmission dynamics of TB in rural areas (Hinggan League [HL], Inner Mongolia) in China. Nearly half of the enrolled TB cases were attributed to recent transmission, a proportion higher than that observed in other rural areas in China (31.4%), highlighting the significance of recent transmission in driving the TB epidemic in this region. Only 19.6% of all drug-resistant TB (DR-TB) cases were found within putative transmission clusters, indicating a lower proportion compared with the previous studies, which indicated that DR-TB is more associated with the *de novo* evolution of resistance within patients. Spatial analysis showed that the TB epidemic was concentrated in densely populated areas in eastern HL. The findings identified epidemiological differences within HL, highlighting the importance of targeted interventions and surveillance to control the spread of TB in HL.

## INTRODUCTION

Tuberculosis (TB), caused by the bacillus *Mycobacterium tuberculosis* (MTB), remains a significant public health challenge. In 2022, TB was the second leading cause of death from a single infectious agent worldwide, following coronavirus disease (COVID-19) (1). According to the World Health Organization (WHO), an estimated 10.6 million people were infected with TB in 2022, of which an estimated 1.6 million died. The high transmission rate combined with the emergence of drug-resistant TB (DR-TB) poses significant threats to TB eradication efforts.

Despite significant progress in TB control in recent years, China continues to bear one of the highest TB burdens globally, with an estimated 748,000 new TB cases (1). Therefore, understanding the transmission dynamics of TB and its associated risk factors is crucial for effective TB control and accurately targeted interventions. Whole-genome sequencing (WGS) has been considered an ultimate tool for genotyping MTB strains, with current protocols providing genomic information from about 90% of the genome (2). While genomic epidemiological approaches have been used to characterize TB transmission dynamics in some developed provinces and specific cities in China, there are few reports on transmission in less developed rural areas with low population densities (3–6).

Inner Mongolia, located in northern China, is a vast multi-ethnic region and one of the provinces with a high TB burden in China (7). Hinggan League (HL) is situated in the northeastern part of Inner Mongolia, with a total area of 55,130 square kilometers (km^2^) and a population density of 29/km^2^. It is reported that TB incidence has consistently ranked first in the province (8). However, information on the transmission patterns and dominant lineages of MTB strains circulating in this region is minimal, which hinders the development and implementation of effective prevention and control strategies.

Therefore, we conducted a retrospective study of MTB strains collected from 2021 to 2023 in HL using WGS. Our study will provide an important basis for understanding the recent transmission patterns of TB in HL, identifying pivotal risk factors, and pinpointing geographical areas of heightened risk. Our findings should support the development of precisely targeted public health interventions.

## MATERIALS AND METHODS

### Study design and population

This retrospective investigation was based on ongoing drug-resistant tuberculosis surveillance work in HL. The study population comprised all MTB strains from presumptive pulmonary TB cases with sputum smear-positive result, who attended local TB designated hospitals in the six administrative subdivisions, Arxan City (AC), Ulanhot City (UC), Jalaid Banner (JB), Tuquan County (TC), Horqin Right Front Banner (HRFB), and Horqin Right Middle Banner (HRMB) in HL between June 2021 and June 2023. All designated hospitals for presumptive pulmonary TB carried out chest X-ray or computed tomography (CT) examination, smear microscopy, and GeneXpert molecular testing. All smear-positive patients and smear-negative patients with pulmonary TB lesions in chest X-ray or CT examination reports would undergo sputum culture. Demographic information (including sex, age, residence, occupation, and nationality) and medical records (including complications and previous TB treatment history) of these TB patients were extracted and matched from the surveillance database. The data were collected and stored electronically by local medical staff during patient visits, following the acquisition of informed consent from the patients.

### Drug susceptibility testing (DST)

Drug susceptibility testing was conducted using UKMYC6 plates (Thermo Fisher Scientific, USA) developed by the CRyPTIC Consortium. UKMYC6 allows for the quantitative assessment of resistance levels to various anti-TB drugs with reliable reproducibility (9). All procedures were carried out by trained and specialized personnel, strictly following prescribed protocols (10). Plates inoculated with the bacterial solution were securely sealed with adhesive seals and then incubated at 37°C in 5% CO_2_ for 14 days. The minimum inhibitory concentration (MIC) was determined as the lowest concentration without evident visible bacterial growth compared with the positive control. The concentration range and breakpoint concentration for each drug on the UKMYC6 plate can be found in Table S1 (11). MTB strains were classified as resistant (R) to a particular drug if the MIC value exceeded the breakpoint concentration.

### WGS and bioinformatics

The genomic DNA was extracted to establish pair-end libraries with a target length of 150 bp, followed by sequencing via the Illumina Hiseq X10 (Illumina, Inc.) with 2 × 150 paired-end (PE) strategies. To specifically identify the *Mycobacterium tuberculosis* (MTB) sequences, we used Kraken with the prebuilt MiniKraken DB_8GB database and identified those isolates as MTB only when a minimum of 90% of reads were mapped to the *M. tuberculosis* complex (12, 13). To guarantee the quality and integrity of the following analysis, the raw FASTQ files were firstly pre-processed with fastp by trimming those low-quality regions to ensure an average read quality of Q20 (14).

In order to identify genetic variations, the filtered reads were aligned with the reference genome H37Rv (GenBank: NC000962.3) using BWA-MEM with default parameters (15). After genome alignment, SAMtools and Genome Analysis Toolkit v4 (GATK4) were used to process the reads (16, 17). As part of this process, bases were recalibrated and realigned to eliminate any potential artifacts. We then exploited the SAMtools/BCFtools suite to identify high-confidence single nucleotide polymorphisms (SNPs) in loci with fixed alternate alleles (frequency >90%) and supported by at least five forward and reverse reads (18). The TB-Profiler v5.0.1 was used to help us determine the MTB lineage and mutations associated with drug resistance (19).

### Phylogenetic analysis

A concatenated alignment was created using fixed SNPs excluding those found in drug resistance-associated genes, polymorphic GC-rich proline-glutamic acid sequences, or proline-glutamic acid sequences (20). Based on this concatenated alignment, maximum-likelihood phylogenetic trees were constructed using IQ-Tree v2.2.2 for all MTB isolates. As part of the tree construction procedure, the following parameters were used: “-m TEST -B 1000,” which represent the automatic model selection used by jModelTest, as well as 1,000 ultrafast bootstrap replicates. With *Mycobacterium canettii* (RefSeq: NC_015848.1) as the outgroup, the highest-scoring ML tree was rooted, and the Interactive Tree of Life (iTOL) was used to visualize the results (21).

### Spatial, temporal, and statistical analysis

A Chinese-language-based web geocoding application tool, supplied by Baidu Maps (Baidu, Beijing, China), was used to geocode the residential addresses of TB patients at the time of diagnosis. The distHaversine function from the R package geosphere was utilized to compute the geographic distances between the geocoded locations (22). To find patterns of spatial aggregation among the enrolled TB patients, two-dimensional (2D) kernel density estimation was conducted using the R package MASS (23). The temporal distribution of diagnosis dates for clustered and unique patients was analyzed using kernel density estimation in R. The R package gtsummary was used to carry out statistical analyses (24). The relationship between genomic clustering was evaluated using the χ test. Fisher’s exact test was used when the sample size was insufficient (i.e., more than 20% of cells had expected frequencies < 5). A P-value of less than 0.05 indicated that a factor’s association with genomic clustering was statistically significant. The R packages ggplot2 and ggpubr were utilized to visualize the outcomes of spatial and statistical analyses (25, 26).

## RESULTS

### Demographic and phenotypic characteristics of TB cases

**Fig 1:**
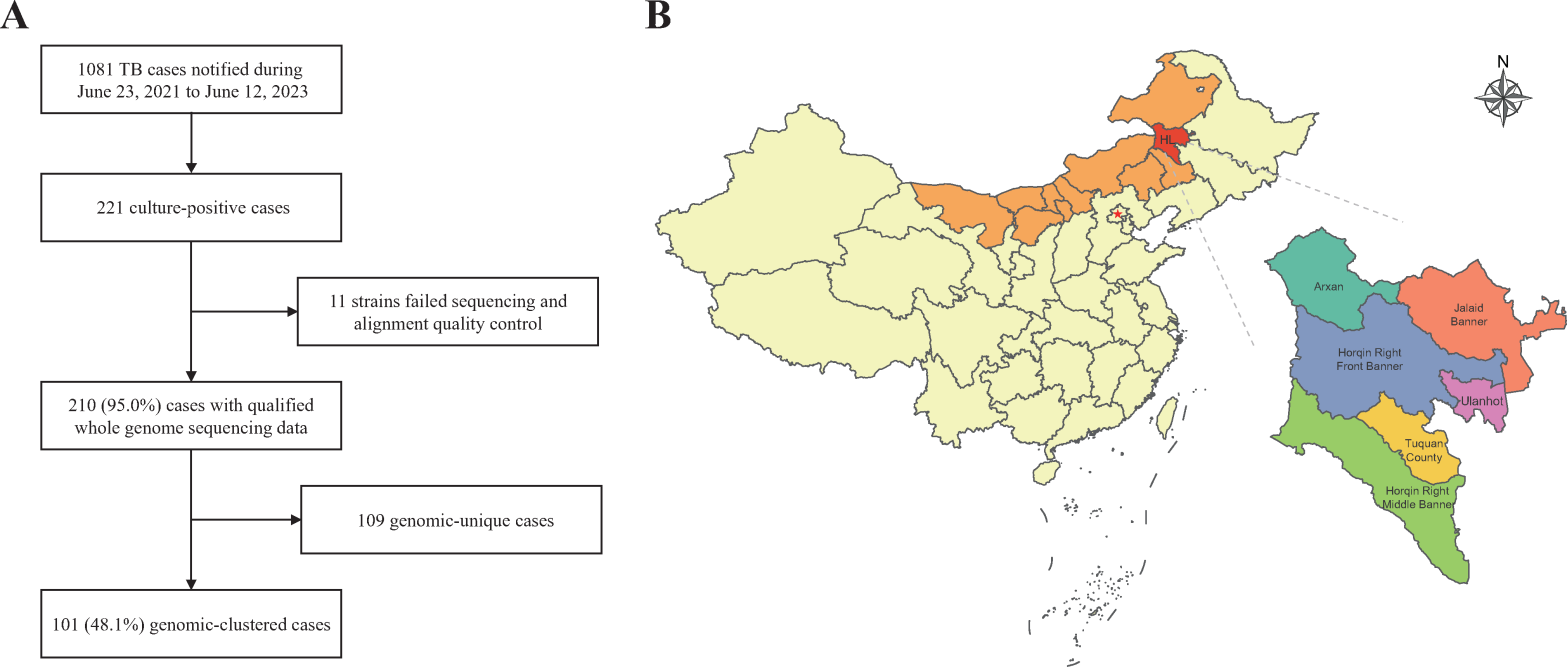
(A) Patient enrollment and study flowchart. (B) Map of the study site in Hinggan League, China. The study site is located in the northeast of Inner Mongolia, China, which includes six administrative subdivisions.

From June 23, 2021, to June 12, 2023, a total of 1,081 TB cases were notified, and 221 (20.4%) clinical MTB strains were isolated from the Hinggan League (HL) of the Inner Mongolia Autonomous Region, China, and successfully sequenced. After quality control, 210 cases were enrolled (Fig. 1). Cases from all six cities and counties within HL were enrolled, with the highest number (51.4%, *n* = 108) in JB, followed by UC (16.2%, *n* = 34), HRFB (13.8%, *n* = 29), HRMB (10.5%, *n* = 22), TC (7.6%, *n* = 16), and AC (0.5%, *n* = 1). The median age of the enrolled patients was 52 years (interquartile range [IQR], 17–87), with 70.5% (*n* = 150) of them being middle-aged and elderly patients over 45 years old. The majority of enrolled patients were male (78.1%, *n* = 164), farmers (70.5%, *n* = 148), and from rural areas (72.4%, *n* = 152). Furthermore, new cases accounted for 73.3% (*n* = 154) of all cases.

Phenotypic DST for 13 anti-TB drugs was performed on all 210 sequenced isolates. The antibiotic phenotypic resistance rates, from high to low, were as follows: isoniazid (INH, *n* = 36, 17.1%), rifampicin (RIF, *n* = 18, 8.6%), rifabutin (RFB, *n* = 16, 7.6%), levofloxacin (LFX, *n* = 9, 4.3%), moxifloxacin (MFX, *n* = 6, 2.9%), amikacin (AMK, *n* = 3, 1.4%), kanamycin (KAN, *n* = 3, 1.4%), clofazimine (CFZ, *n* = 3, 1.4%), delamanid (DLM, *n* = 2, 0.1%), ethionamide (ETO, *n* = 2, 0.1%), and linezolid (LZD, *n* = 1, 0.5%). No strains with bedaquiline (BDQ) resistance were found. The overall drug-resistant TB (DR-TB) rate was 21.9% (46/210), with seven RR-TB, 25 HR-TB, six MDR-TB, and five pre-XDR-TB.

### Phylogenetic analysis of MTB strains

**Fig 2:**
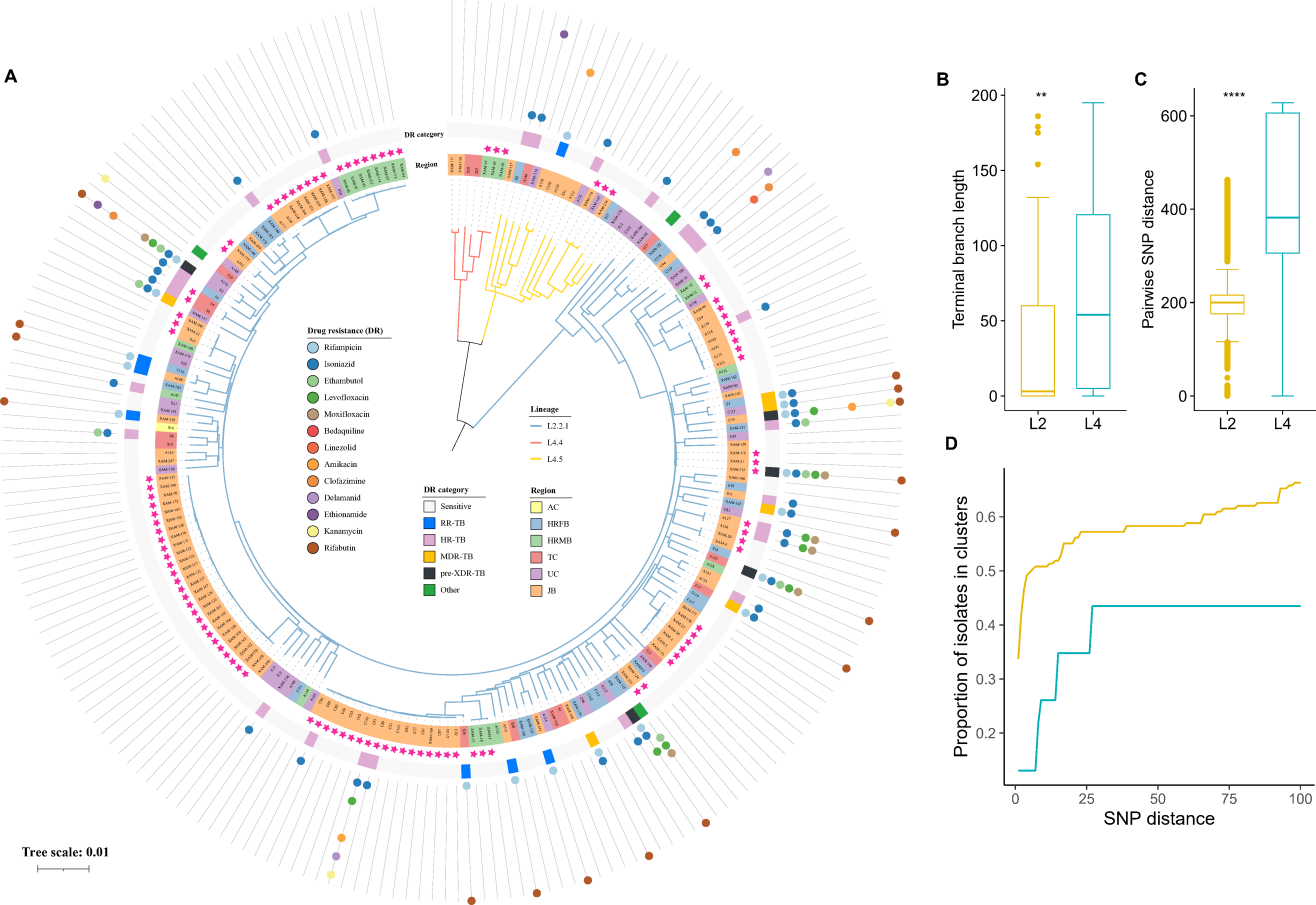
Properties of phylogeny for MTB genomes in HL. (**A**) Phylogenetic tree of 210 sequenced MTB strains and annotated with drug-resistant information. The different colored branch lines indicate the sublineages. The background colors of tip nodes are based on the six administrative subdivisions (regions) where the patient lives. Solid stars outside the phylogeny indicate genomic-clustered strains differing by ≤10 single nucleotide polymorphisms (SNPs). The outer broken colored circle indicates the phenotypic DR category. The outermost colored dots indicate the phenotypic resistance to the 13 anti-TB drugs tested. Distributions of terminal branch lengths (**B**) and pairwise SNP distances (**C**) for the L2 and L4 phylogeny. (**D**) Proportion of isolates from L2 and L4 that belong to clusters (*y*-axis) defined at different thresholds for maximum pairwise SNP distances (*x*-axis).

To elucidate the genetic structures of the enrolled MTB strains, we reconstructed a maximum likelihood (ML) phylogenetic tree using concatenated sequences derived from non-redundant SNP loci of 210 strains (Fig. 2A). Genotyping analysis based on TB-profiler identified that 89.0% (*n* = 187) of the strains belonged to sub-lineage 2.2.1 in lineage 2 (L2), commonly referred to as the Beijing family. The remaining 11.0% (23/210) of the strains belonged to lineage 4 (L4), comprising two subfamilies: L4.5 (*n* = 16) and L4.4.2 (*n* = 7), respectively.

To compare the transmissibility and disease-causing potential between L2 and L4 strains in HL, we analyzed diversity metrics for their respective phylogenetic subtrees. Compared with L4, L2 strains showed significantly shorter terminal branch lengths (Fig. 2B, Wilcoxon test *P* < 0.001) and greater genetic similarity, with a smaller median pairwise SNP distance (Fig. 2C, Wilcoxon test *P* < 0.001). We further investigated transmission dynamics by examining the distribution of potential genomic clusters using various maximum pairwise SNP distance thresholds (1-100 SNPs) to define clusters (Fig. 2D). The proportion of strains in genomic clusters was higher among L2 strains compared to L4 strains.

### Identification of genomic-clustered cases and their risk factors

Transmission clusters were defined as strains differing by a maximum of 10 SNPs. A total of 101 strains were grouped into 19 genomic clusters (Table S2), resulting in a clustering rate of 48.1% (101/210). The largest cluster (C1) contained 26 strains, followed by C2 with 18 strains, C3 with eight strains, and the remaining clusters had varying numbers of members ranging from 2 to 6.

Nine phenotypic DR-TB strains were identified in five genomic clusters (C2, C4, C9, C14, and C16). C2, C14, and C16 might indicate recent transmission of DR-TB, as they exhibited identical isoniazid resistance mutations within each cluster. Additionally, mutations associated with fluoroquinolones and/or pyrazinamide were consistently found in all DR-TB strains within two clusters (C14 and C2). Concerningly, one strain (XAM-151) in C9 had resistance mutations associated with rifampicin (rpoB Ser450Leu), isoniazid (ahpC c.-48G > A and fabG1 c.-8T > A), ethambutol (embB p.Met306Val), and fluoroquinolones (gyrA p.Asp94Ala), while the other two members of the cluster were pan-susceptible. XAM-151 was isolated from a treatment-naïve patient with tuberculous pleurisy.

To identify risk factors associated with genomic-clustered cases, demographic and clinical characteristics of patients, as well as the genetic background of MTB strains, were included in the logistic regression model. The results indicate a significant correlation between the lineage and drug resistance of strains and the geographic location of patients with genomic clustering (Table 1). Multivariate analysis revealed that recent TB transmission was more likely to occur in patients infected with L2 strains compared with L4 strains (51.3% vs 26.1%, *P* = 0.016) and those living in JB (75.9% vs 18.6%, *P* < 0.001). DR-TB strains were less frequent in clusters than in non-clusters (8.9% vs. 33.9%, *P* < 0.001).

**TABLE 1:** Univariate and multivariate analyses of risk factors related to genomic clustering^*a*^

Variables	Clustering (*n* = 101, rate[%])	Cor (95% CI)	P	Aor (95% CI)	*P*
Age (years)			0.9		
<30	7 (43.8)	0.733 (0.247–2.181)			
30–44	20 (45.5)	0.787 (0.368–1.665)			
45–59	37 (47.4)	0.852 (0.449–1.616)			
≥60	37 (51.4)	Ref			
Sex			0.1		0.3
Female	27 (58.7)	1.733 (0.896–3.32)		1.537 (0.651–3.669)	
Male	74 (45.1)	Ref		Ref	
Area			<0.001		**<0.001**
Jalaid Banner	82 (75.9)	13.464 (7.389–27.113)		14.88 (7.389–29.964)	
Others	19 (18.6)	Ref		Ref	
Occupation			0.89		
Farmer	73 (49.3)	1.041 (0.566–1.916)			
Non-farmer	28 (48.3)	Ref			
Nationality			0.51		
Han	50 (50.5)	1.197 (0.698–2.075)			
Others	51 (45.9)	Ref			
EPTB			0.89		
Yes	8 (50)	1.073 (0.383–3.004)			
No	92 (48.2)	Ref			
Diabetes			0.89		
Yes	8 (50)	1.073 (0.383–3.004)			
No	92 (48.2)	Ref			
Treatment history			0.74		
Yes	28 (50)	1.105 (0.6–2.054)			
No	73 (47.4)	Ref			
Residence			0.97		
Rural	73 (48)	0.99 (0.538–1.822)			
Urban	28 (48.3)	Ref			
Lineage			0.022		**0.016**
L2	95 (50.8)	3.004 (1.162–8.166)		4.055 (1.297–13.464)	
L4	6 (26.1)	Ref		Ref	
Phenotypic DR type			<0.001		**<0.001**
Pan-susceptible	92 (56.1)	5.474 (2.484–12.182)		5.474 (2.138–14.88)	
DR-TB	9 (19.6)	Ref		Ref	

^*a*^ Note: COR, crude odds ratio; AOR, adjusted odds ratio; CI, confidence interval; *, not included in univariate and multivariate logistic regression models; variables with *P* < 0.2 at univariate analysis were included in multivariate logistic regression model.

### Spatial distribution of TB cases

**Fig 3:**
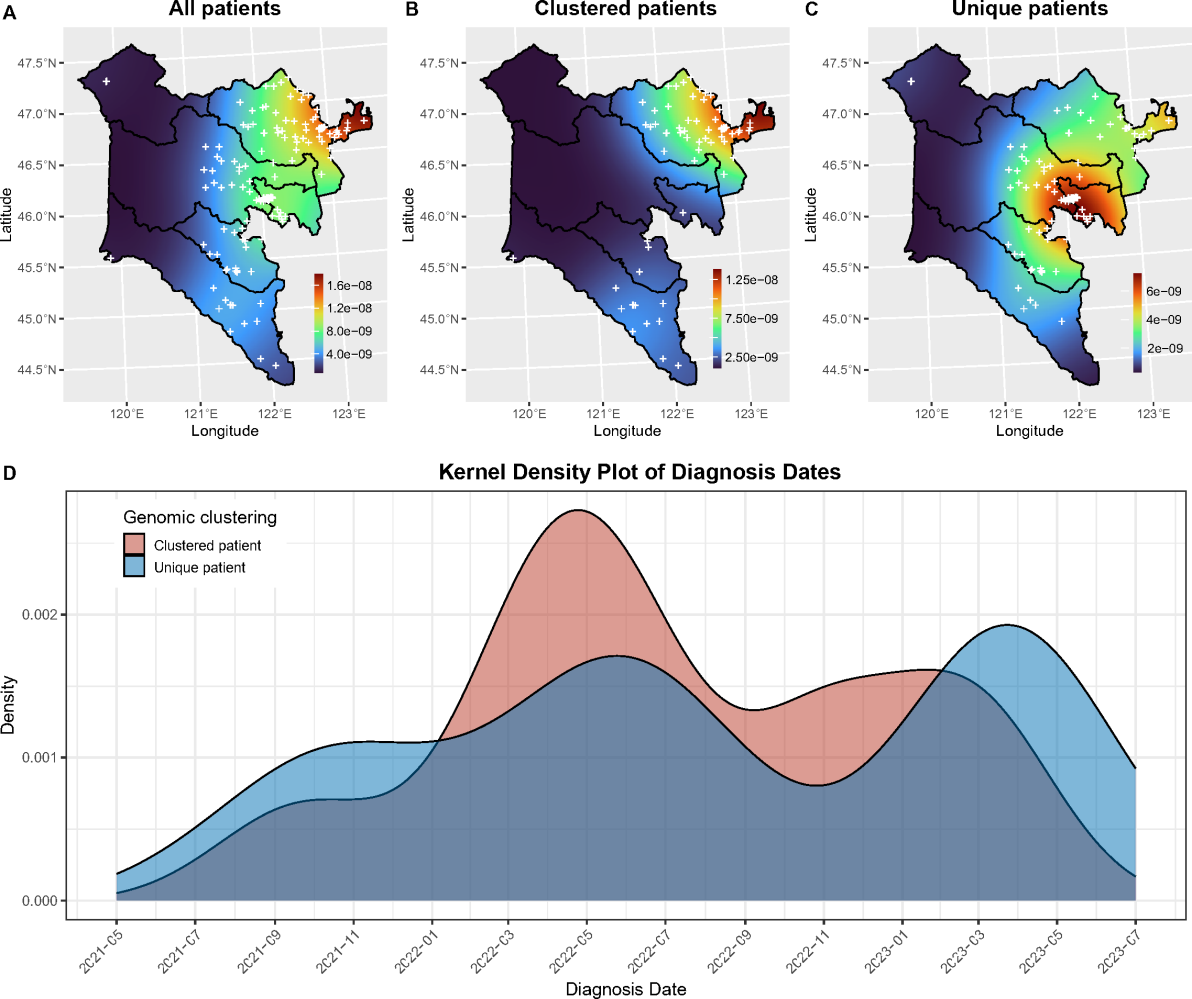
Spatial and temporal distribution of TB cases in HL. Kernel density estimation of the residential locations for all 210 patients (**A**), clustered patients (**B**), and unique patients (**C**). Cross symbols represent individual patient residences. (**D**) Kernel density plot showing the temporal distribution of diagnosis dates, estimated using a Gaussian kernel to smooth the frequency of cases over time.

To enhance our understanding of the spatial distribution, kernel density maps were generated using the residential addresses of all patients. The majority of enrolled TB cases in HL were concentrated in the northeast of the region (Fig. 3A). A notable hotspot for the TB epidemic was identified in the northeastern part of HL, specifically in the urban center of JB. Analysis of the residential addresses of 101 patients carrying clustered strains (Fig. 3B) revealed that the hotspot for recent transmission was also located in the urban center of JB. In contrast, unique cases were concentrated in the central part of HL, including the city center of UC and its adjacent area of HRFB (Fig. 3C). Only four and one clustered strains were identified from patients living in TC and UC, respectively, with three of them also belonging to the same transmission chains as the cases in JB and HRMB. All strains in AC and HRFB were unique.

Furthermore, 18 out of 19 genomic clusters (94.7%) were confined to a single region, with only cluster 10 containing cases from both JB and UC (Fig. S1). Upon examining the residential addresses within each genomic cluster, we determined that the median geographical distance between clustered cases was 49 (range, 0 to 218) km. The distance did not change significantly when adjusting the threshold for defining genomic clusters from 12 single nucleotide polymorphisms (SNPs) to five SNPs or one SNP (*P* = 0.51 and *P* = 0.09, by Wilcoxon rank-sum test; Fig. S2). Defining living in the same community as residing within a 2-km radius, only 29 out of 101 patients (28.7%) with clustered strains were identified as living in the same community, and no genomic cluster contained only cases all residing in the same community.

The kernel density estimation plot of diagnosis dates revealed a similar periodic fluctuation pattern for both clustered and unique patients (Fig. 3D). Specifically, the diagnosis rate increased in the first half of each year and declined in the second half. Notably, the diagnosis density of clustered cases was higher than that of unique cases in 2022 (0.00273 vs 0.00171), whereas in 2021 (0.00103 vs 0.00111) and 2023 (0.00161 vs 0.00193), the density of clustered cases was relatively lower.

## DISCUSSION

TB remains a major public health problem in China, although the population characteristics and DR profiles in different regions vary greatly (27). A previous study illustrated that more than 25% of MTB strains exhibited resistance to at least one anti-TB drug tested, highlighting a relatively severe drug-resistant situation in HL (28). However, the transmission of MTB, especially the transmission mode of DR-TB in HL, has not been studied, so in this study, we constructed the transmission dynamics of MTB in HL, Inner Mongolia, China. The study found nearly half (48.1%) of the enrolled TB cases were attributed to recent transmission, while only 19.6% of all DR-TB cases were found within putative transmission clusters.

WGS-based genotyping demonstrated a high prevalence of L2-Beijing strains (89.0%) among TB cases in HL. This finding is consistent with previous research that reports the Beijing genotype as predominant in several Chinese regions, including Tibet (90.63%), Jilin (89.9%), Heilongjiang (89.5%), and Shanghai (89%) (28–30). The widespread prevalence of the Beijing genotype may be due to its high transmission efficiency, increased virulence, and elevated drug resistance risk (31). It has been suggested that the mutation rate for L2-Beijing strains is higher than for L4 strains (32). This means that if all MTB lineages had similar transmission dynamics in the study area, we would expect longer terminal branch lengths for L2-Beijing strains. However, our observations indicate the opposite. Therefore, we conclude that the higher frequency of L2-Beijing strain infections and their shorter branch lengths imply more recent ongoing transmission of L2 strains compared with other strains within the study population.

WGS methods can be effectively combined with epidemiological data to study the transmission dynamics of MTB strains. In HL, nearly half (48.1%) of the enrolled TB cases were attributed to recent transmission, a proportion higher than that observed in other rural areas in China (31.4%), highlighting the significance of recent transmission in driving the TB epidemic in this region (4). Among the included patients, only 27% were previously retreated patients, but the proportion of patients in the cluster is similar for both new and previously treated cases, suggesting that previously retreated patients are more likely to cause recent transmission. Adherence to treatment should be ensured in new and retreatment cases to increase cure rates and reduce transmission of TB in the community. Additionally, only 19.6% of all DR-TB cases were found within putative transmission clusters, indicating a lower proportion compared with the 81.4% of MDR-TB cases likely due to the transmission of MDR strains (4). Moreover, we only observed seven DR-TB strains within three clusters, which might indicate recent transmission of DR-TB, which indicated that DR-TB is more associated with the *de novo* evolution of resistance within patients.

Spatial analysis showed that the TB epidemic was concentrated in densely populated areas in eastern HL, including JB, UC, and the eastern part of HRFB bordering UC. UC is the traditional central urban area of HL, with an urbanization rate of 89.6% in 2022. In comparison, JB is a typical rural area, with an urbanization rate of only 45.5%. Notably, the epidemiology of new TB cases differed significantly among these areas, with JB primarily experiencing recent transmission, while UC and HRFB likely faced endogenous resurgence of latent infection. This information suggests that different TB prevention and control strategies should be targeted in urban and rural areas of HL. In JB, where recent transmission is prevalent, efforts should focus on identifying sources of infection and interrupting transmission routes (33). In contrast, for UC, where endogenous reactivation is more common, the emphasis should be on establishing flexible preventive treatment policies to protect individuals at high risk of *M. tuberculosis* infection and active disease development (34). Furthermore, our data showed patients within the cluster in HL appeared to be living distantly, which indicated that the transmission of TB in HL seemed to be countywide with large distances.

The temporal distribution of TB cases in HL showed a seasonal pattern, with diagnosis rates peaking in the first half of each year and declining in the second half. Both clustered and unique cases exhibited this trend, suggesting potential influences from climate, healthcare-seeking behavior, or seasonal variations in transmission. Notably, the diagnosis density of clustered cases was higher in 2022 but lower in 2021 and 2023, which may indicate a localized outbreak or increased transmission of genetically related strains. Possible contributing factors include changes in surveillance efforts, hospital detection capacity, or variations in social interactions, warranting further investigation.

In conclusion, this study provides valuable insights into the molecular epidemiology and transmission dynamics of TB in HL, Inner Mongolia, China. The findings identified epidemiological differences within the region studied, highlighting the importance of targeted interventions, drug resistance management, and surveillance to control the spread of TB in the region. Further research is needed to validate these findings and explore additional factors influencing TB transmission and drug resistance in HL.

## Data Availability

The raw sequence data reported in this paper have been deposited in the Genome Sequence Archive (Genomics, Proteomics & Bioinformatics 2021) in National Genomics Data Center (Nucleic Acids Res 2022), China National Center for Bioinformation/Beijing Institute of Genomics, Chinese Academy of Sciences (GSA: CRA017087) that are publicly accessible at https://ngdc.cncb.ac.cn/gsa.

## SUPPLEMENTAL MATERIAL

Fig. S1 spectrum.00169-25-s0001.pdf Spatial distribution of case residences (red dots) across the 19 identified genomic clusters.

Fig. S2 spectrum.00169-25-s0002.pdf Residential distance distribution of clustered sample pairs across SNP divergence thresholds (1, 5, 12, 50). Red dashed line shows median distance.

Supplemental material spectrum.00169-25-s0003.docx Legends for supplemental material.

Table S1 spectrum.00169-25-s0004.docx Concentration range and breakpoints of anti-TB drugs involved in this study.

Table S2 spectrum.00169-25-s0005.xlsx *Mycobacterium tuberculosis* isolates in this study.

ASM does not own the copyrights to Supplemental Material that may be linked to, or accessed through, an article. The authors have granted ASM a non-exclusive, world-wide license to publish the Supplemental Material files. Please contact the corresponding author directly for reuse.
