# Supplementary material for: High proportion of tuberculosis recent transmission in rural areas of Northeastern China: a 3-year prospective population-based genotypic and spatial analysis in Hinggan League, China
Source: Microbiol Spectr. 2025 Jul 11;13(8):e00169-25. doi: 10.1128/spectrum.00169-25 (PMC12323342; doi:10.1128/spectrum.00169-25)
Supplement: Fig. S1 — Spatial distribution of case residences (red dots) across the 19 identified genomic clusters. [file spectrum.00169-25-s0001.pdf]

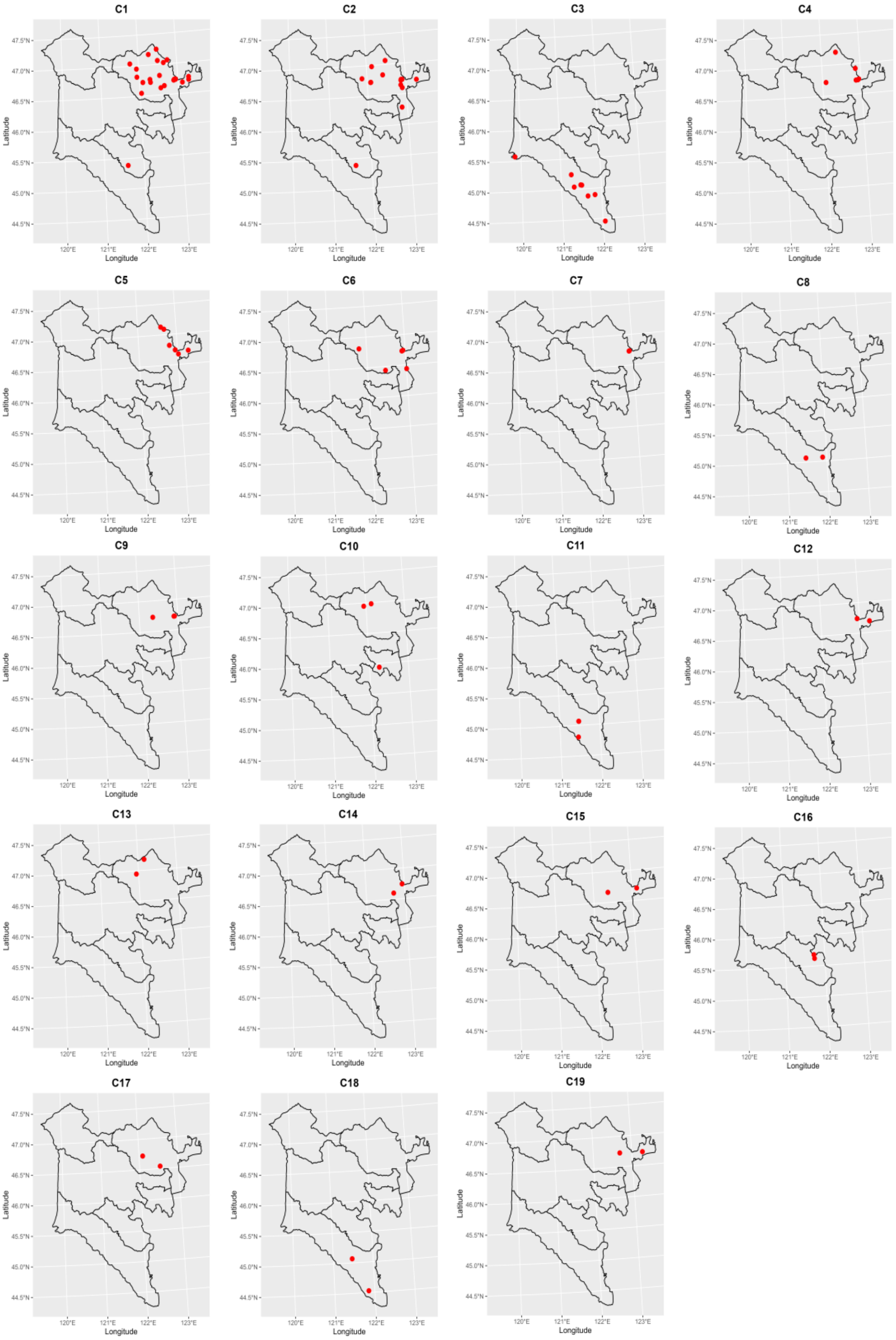

Figure S1. Spatial distribution of case residences (red dots) across the 19 identified genomic clusters.
