# Supplementary material for: High proportion of tuberculosis recent transmission in rural areas of Northeastern China: a 3-year prospective population-based genotypic and spatial analysis in Hinggan League, China
Source: Microbiol Spectr. 2025 Jul 11;13(8):e00169-25. doi: 10.1128/spectrum.00169-25 (PMC12323342; doi:10.1128/spectrum.00169-25)
Supplement: Fig. S2 — Residential distance distribution of clustered sample pairs across SNP divergence thresholds (1, 5, 12, 50). Red dashed line shows median distance. [file spectrum.00169-25-s0002.pdf]

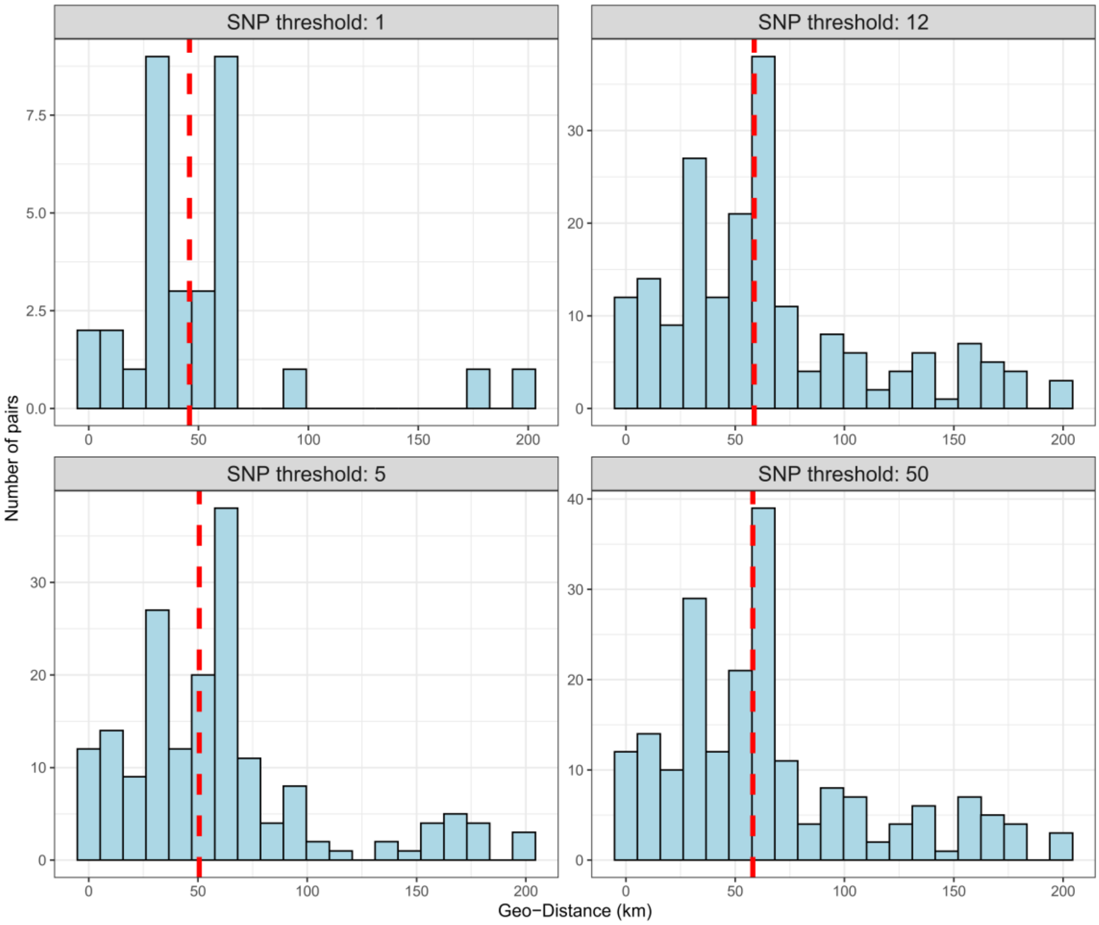

Figure S2. Residential distance distribution of clustered sample pairs across SNP divergence thresholds (1, 5, 12, 50). Red dashed line shows median distance.
