# Supplementary material for: High proportion of tuberculosis recent transmission in rural areas of Northeastern China: a 3-year prospective population-based genotypic and spatial analysis in Hinggan League, China
Source: Microbiol Spectr. 2025 Jul 11;13(8):e00169-25. doi: 10.1128/spectrum.00169-25 (PMC12323342; doi:10.1128/spectrum.00169-25)
Supplement: Supplemental material — Legends for supplemental material. [file spectrum.00169-25-s0003.docx]

Figure S1. Spatial distribution of case residences (red dots) across the 19 identified genomic clusters.​

Figure S2. Residential distance distribution of clustered sample pairs across SNP divergence thresholds (1, 5, 12, 50). Red dashed line shows median distance.

Table S1. Concentration range and breakpoints of anti-TB drugs involved in this study.

Table S2. The information of *Mycobacterium tuberculosis* isolates in this study.
