# Supplementary material for: High proportion of tuberculosis recent transmission in rural areas of Northeastern China: a 3-year prospective population-based genotypic and spatial analysis in Hinggan League, China
Source: Microbiol Spectr. 2025 Jul 11;13(8):e00169-25. doi: 10.1128/spectrum.00169-25 (PMC12323342; doi:10.1128/spectrum.00169-25)
Supplement: Table S1 — Concentration range and breakpoints of anti-TB drugs involved in this study. [file spectrum.00169-25-s0004.docx]

Table S1. Concentration range and breakpoints of anti-TB drugs involved in this study

| Drug | Drug abbr. | Concentration range | Breakpoint (μg/mL)^1^ |
| --- | --- | --- | --- |
|  |  | (μg/mL) |  |
| Isoniazid | INH | 0.025-12.8 | 0.2 |
| Rifampin | RIF | 0.03-8 | 1 |
| Rifabutin | RFB | 0.06-2 | 0.5 |
| Ethambutol | EMB | 0.25-32 | 5 |
| Amikacin | AMI | 0.25-16 | 4 |
| Kanamycin | KAN | 1-16 | 5 |
| Levofloxacin | LFX | 0.12-8 | 1 |
| Moxifloxacin | MXF | 0.06-4 | 0.5 |
| Ethionamide | ETH | 0.25-8 | 5 |
| Delamanid | DLM | 0.008-0.5 | 0.2 |
| Bedaquiline | BDQ | 0.008-1 | 0.25 |
| Linezolid | LZD | 0.06-4 | 1 |
| Clofazimine | CFZ | 0.03-2 | 1 |
| ^1^ CRyPTIC Consortium. Epidemiological cut-off values for a 96-well broth microdilution plate for high-throughput research antibiotic susceptibility testing of M tuberculosis. Eur Respir J 2022; 60: 2200239. | | | |
